# In vivo fluorescence molecular tomography of induced haemarthrosis in haemophilic mice: link between bleeding characteristics and development of bone pathology

**DOI:** 10.1186/s12891-020-03267-5

**Published:** 2020-04-14

**Authors:** K. K. Vøls, M. Kjelgaard-Hansen, C. D. Ley, A. K. Hansen, M. Petersen

**Affiliations:** 1 Global Drug Discovery, Novo Nordisk A/S, Novo Nordisk Park 1, 2760 Maaloev, Denmark; 2 Veterinary and Animal Sciences, University of Copenhagen, Frederiksberg, Denmark; 3 Veterinary Clinical Sciences, University of Copenhagen, Frederiksberg, Denmark

**Keywords:** Haemophilia a, Haemophilic arthropathy, Mouse model, In vivo imaging, Fluorescence molecular tomography, Micro-CT

## Abstract

**Background:**

Haemophilic arthropathy is a chronic and debilitating joint disease caused by recurrent spontaneous joint bleeds in patients with haemophilia. Understanding how characteristics of individual joint bleeds relate to the subsequent development of arthropathy could improve management and prevention of this joint disease. Here, we aimed to explore relations between joint bleed characteristics and development of bone pathology in a mouse model of haemophilic arthropathy by using novel in vivo imaging methodology.

**Methods:**

We characterised induced knee bleeds in a murine model of haemophilic arthropathy by quantitative in vivo fluorescence molecular tomography (FMT) and by measurements of changes in the diameter of the injured knee. Wild-type mice and non-injured haemophilic mice acted as controls. Development of arthropathy was characterised by post mortem evaluation of bone pathology by micro-CT 14 days after bleed-induction. In an in vitro study, we assessed the effect of blood on the quantification of fluorescent signal with FMT.

**Results:**

In most injured haemophilic mice, we observed significant loss of trabecular bone, and half of the mice developed pathological bone remodelling. Development of pathological bone remodelling was associated with significantly increased fluorescent signal and diameter of the injured knee just 1 day after induction of the bleed. Further, a correlation between the fluorescent signal 1 day after induction of the bleed and loss of trabecular bone reached borderline significance. In the in vitro study, we found that high concentrations of blood significantly decreased the fluorescent signal.

**Conclusion:**

Our results add novel insights on the pathogenesis of haemophilic arthropathy and underline the importance of the acute phase of joint bleeds for the subsequent development of arthropathy.

## Background

Lack of coagulation factor VIII (FVIII) activity makes patients with haemophilia A unable to clot normally [1]. As a consequence, patients with severe haemophilia A (< 1% FVIII activity) experience spontaneous joint bleeds [2]. Upon bleeding, patients may develop ‘target joints’ that are characterised by synovial proliferation and angiogenesis. This predisposes the joint to repetitive bleeding, ultimately resulting in a vicious cycle in which more and more bleeds occur in the same joint [3–6]. Over time, the entire joint may be affected, including pathological manifestations such as haemophilic synovitis, cartilage degradation, osteopenia and bone remodelling [4, 7].

The exact pathogenesis leading to joint destruction is not fully understood, and protection against arthropathy remains incomplete despite prophylactic treatment with recombinant FVIII [8, 9]. Although the number of radiologically-evident joint changes have been reported to increase with increasing number of clinically-evident bleeds [10, 11], there are sub-populations of patients in which very few (or even 0) clinically-evident joint bleeds are associated with a high degree of arthropathy [12] that may be progressive despite intensive prophylaxis [13]. It has been suggested that degenerative osteochondral changes may be initiated if the amount of blood in the joint has exceeded a certain threshold, even for a short time [14]. However, none of the above-mentioned clinical studies account for the severity of the individual joint bleeds, leaving it unknown if certain characteristics of the joint bleeds are critical for the development of arthropathy, and in particular bone pathology.

To study blood-induced joint damage, haemophilic rodent models have been used extensively [15]. Upon induced joint bleeding, the FVIII knock out (F8-KO) mouse develops joint damage comparable to the human disease as evaluated by histology [16]. In order to better understand how the disease progresses from bleeding to arthropathy, in vivo imaging (ultrasound, micro-computed tomography (micro-CT) and positron emission tomography/CT) has recently been applied in studies with haemophilic mice [5, 17–19]. Potentially, in vivo optical fluorescent imaging could be well-suited to study blood-induced joint damage, as several aspects of the pathogenesis can be visualised and described simultaneously using non-targeted, targeted or activity-based imaging, depending on the fluorophore applied [20, 21]. Specifically, non-targeted fluorescent blood pool agents could be used as markers for blood and hence used to longitudinally characterise induced bleeds. For this purpose, optical imaging with fluorescence molecular tomography (FMT) has the advantages of being quantitative, highly sensitive and with a relatively high imaging depth as compared to other optical imaging modalities like confocal- and two-photon microscopy or fluorescence reflectance imaging [22–24].

In this study, we aimed to characterise and quantify induced joint bleeds in haemophilic mice in vivo with FMT, in order to investigate potential relations between bleeding characteristics and the subsequent development of bone pathology.

## Methods

### Study design

Haemophilia A (B6; 129S4-F8^tm1Kaz^, Taconic, Lille Skensved, Denmark; F8-KO) and wild type (C57BL/6 N, Taconic, Lille Skensved, Denmark; WT) mice were dosed with a fluorescent blood pool agent prior to induced joint bleed in the left knee. A sub-population of the F8-KO mice acted as shams, meaning no joint bleed was induced. Subsequently, mice were imaged with FMT four times in the following 2 days to characterise the bleed. After 14 days, the mice were euthanised by cervical dislocation while under anaesthesia, and the knees imaged with micro-CT to evaluate bone pathology.

### Animals

Three months old F8-KO (*n* = 35) and WT (*n* = 7) mice of both sexes were included in the study and group housed under standard conditions: 12/12 h light-dark cycle, ad libitum water and ad libitum low fluorescent chow (Altromin C1039, Altromin®, Lage, Germany). All animal procedures were performed under inhalation anaesthesia (1.6–5% isoflurane, 0.7 L/min N_2_O, 0.3 L/min O_2_), providing fast induction and recovery from anaesthesia. As this was an exploratory study based on novel methodology, group sizes were calculated based on the incidence of bone pathology seen in this haemophilic murine model after a single induced joint bleed in previous studies in our lab.

The day before the study, the imaged area containing the knee joints was shaved, and a depilatory cream (Veet® Green, Reckitt Benckiser Group, Slough, UK) applied to the skin for 1 min before being washed off. Extra nesting material was provided in the cages throughout the study.

Buprenorphine analgesia (Temgesic®, Indivior UK Limited, Berkshire, UK) – that has been shown not to interfere with the pathological outcomes [25] - was provided as repeated subcutaneous doses on the first day (0.1 mg/kg at 0 h, 6 h, 12 h, and 24 h) and in the drinking water (6 mg/L) from 12 h onwards. Mice were subjected to daily welfare observations and humane endpoints.

### Fluorescent blood pool agent

All mice were dosed intravenously (iv, 100 μL/25 g corresponding to 2 nmol/25 g) with AngioSense750 (Perkin Elmer, Waltham, MA, USA) 3 min prior to joint bleed induction. AngioSense750 is a non-immunogenic fluorescent macromolecule designed to have a long half-life (7 h) in the vasculature of small rodents (supplier information, [26]). The excitation and emission maxima are 750 nm and 780 nm, respectively. AngioSense750 can be used to image blood vessels, angiogenesis and vascular changes in relation to arthritis (supplier information).

### Joint bleed induction

Haemarthrosis was induced in the left knee of anaesthetised F8-KO (*n* = 29) and WT mice (*n* = 7). Briefly, the knee was cleansed with an alcohol swab and maintained in a flexed position. A 30G needle was gently advanced approximately 2 mm into the knee joint through the subpatellar ligament, making sure not to damage bone or cartilage. Six randomised F8-KO mice acted as shams for this procedure: the sham procedure consisted of cleansing the knee joint with an alcohol swab [19]. To confirm successful induction of joint bleeding, the diameter of the knee joints was measured prior to induction of joint bleed, and 24 h afterwards. At both time points, the knee diameter was measured five times using a digital caliper (Mitutoyo Corporation, Kawasaki, Japan). The delta diameter (Δdiameter) was calculated by subtracting the mean diameter of the contralateral knee from the mean diameter of the induced knee, and can be interpreted as a change in joint diameter due to inflammation, effusion and bleeding [27].

### In vivo fluorescence molecular tomography

**Fig. 1:**
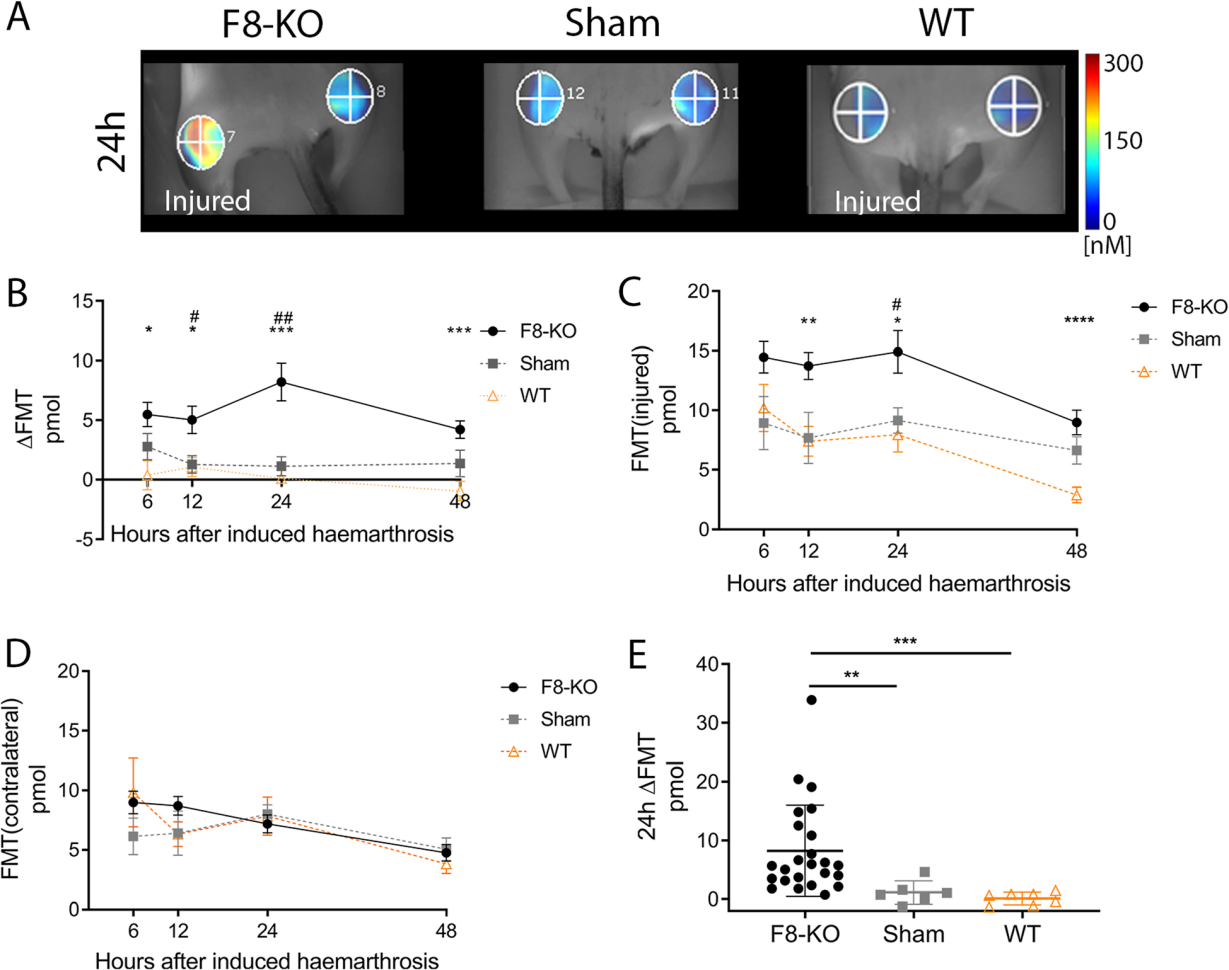
In vivo FMT. **a** FMT scan of an F8-KO, sham and WT mouse imaged 24 h after induced joint bleed. VOIs are drawn around both knee joints; colour-bar indicates signal concentration in the VOI. **b** Relative (ΔFMT) and absolute (**c**) fluorescent signal in the injured (or left; sham) or contralateral (**d**) knees of F8-KO, sham and WT mice over time (mean +/− standard error of the mean). **e**: 24 h ΔFMT in the three groups (mean +/− standard deviation). Statistical differences between F8-KO and sham or WT mice are marked with # or *, respectively. Statistical significance levels are marked: *P < 0.05, **P < 0.01, ***P < 0.001, ****P < 0.0001

Anaesthetised mice were placed in prone position in an FMT animal imaging cassette. The knee joints were pointed laterally and placed on an imaging block (Supplemental Table 1) to enhance the scan area containing the knees (Fig. 1a). Data were acquired by multiple point-source transilluminations with an 80 mW laser (750 nm) using the FMT1500® (Perkin Elmer, Waltham, MA, USA). Each scan included both knee joints and scan time was approximately 7 min.

Correct iv-dosing of AngioSense750 was confirmed by 2D reflectance imaging 3 min after induced joint bleed; images of the tail and superficial vessels of the lower body were evaluated for presence of fluorescence. Mice were not included in the study if iv injection was incomplete. To follow the fluorophore distribution in the knees and establish the optimal imaging time point, imaging with FMT was performed at 6 h, 12 h, 24 h, and 48 h post induction.

### Post mortem micro-CT

On Day 14, the knee joints were imaged post mortem with micro-CT in a Quantum FX® (Perkin Elmer, Waltham, MA, USA) at field of view 10 mm, 20 μm voxel size, 200 ms integration time, 90 kV, and 160 μA.

### Fluorescence molecular tomography analysis

FMT scans were analysed with Truequant 4.0® software (Perkin Elmer, Waltham, MA, USA). Volumes of interest (VOI) of approximately 2500mm^3^ were drawn around both knees. The observer was blinded to the experimental group. The fluorescent signals in the injured and contralateral knees were designated FMT (injured) and FMT (contralateral), respectively. The relative fluorescent signal in the injured knee, ΔFMT, was defined as FMT (injured) subtracted FMT (contralateral) and was used to characterise the extra signal observed in the injured knee, corrected for signal in the contralateral. At two time points, one F8-KO mice could not be measured (software malfunction).

### Micro-CT analysis

Pathological bone remodelling on cortical bone (osteophytosis on femur, patella and tibia; periosteal bone formation on femur and tibia) was scored on a semi-quantitative score (0–5) by reviewing the coronal, sagittal, and transverse plane of the entire scan volume [18]. A score of 0 indicated no pathological changes, whereas a max score of 5 indicated presence of all cortical pathological bone remodelling changes. In addition, 900 μm of the trabecular bone of the tibia, distal to the growth zone was analysed to calculate the trabecular bone volumetric density (BV/TV) and the cortical bone volume, using the BMA module in Analyze 12.0 (AnalyzeDirect, Overland Park, KS, USA). For these analyses, mice were excluded if image positioning was inappropriate (*n* = 2). The observer was blinded to the experimental group.

### In vitro fluorescence molecular tomography

Secondary to our observations of FMT signal intensities in the in vivo study, we performed a post hoc in vitro study to assess whether the presence of large amounts of blood affected the quantification of fluorophore. Briefly, 500 pmol of AngioSense750 was added to an Eppendorf tube filled with either 1.5 mL phosphate buffered saline (PBS); 1.5 mL haemophilic mouse whole blood (WB); or a combination (0.75 mL PBS + 0.75 mL WB). Each setup was repeated 4 times. All samples were imaged by 84 transilluminations. The fluorescence was quantified by drawing a VOI around the entire Eppendorf tube.

### Statistics

Differences between groups with regards to temporal fluorescent signals in the knees were tested with the mixed-effect analysis with Geisser-Greenhouse correction with Tukey’s (3 groups) correction for multiple comparisons. At 24 h, differences in fluorescent signals were tested with Kruskal Wallis test with Dunn’s correction for multiple comparisons (3 groups) or Mann-Whitney (2 groups). Changes in Δdiameter, and BV/TV were tested with the Wilcoxon matched-pairs signed rank test (non-Gaussian distributed data), and changes in cortical bone volume were tested with paired t-test (Gaussian-distributed data). Differences in micro-CT scores for pathological bone remodelling were tested with Kruskal Wallis test with Dunn’s correction for multiple comparisons. For all comparisons, data were tested for Gaussian distribution with the D’Agostino and Pearson test. Correlation strengths were tested with Pearson’s correlation coefficient. Results were considered as significant if *p* < 0.05. Statistical analyses were performed with Prism (version 8.02, GraphPad Software, Inc., San Diego, CA, USA).

## Results

### In vivo characterisation of induced joint bleeds

Twenty-four F8-KO mice, 7 WT and 6 F8-KO sham mice completed the study and were included in the analysis. Five F8-KO mice were euthanised due to humane endpoints. All mice were imaged with FMT at 6 h, 12 h, 24 h, and 48 h post joint bleed induction to temporally characterise the induced bleeds and to determine the optimal imaging time point, at which the ΔFMT (fluorescent signal in the injured knee corrected for signal in the contralateral knee) peaked (Fig. 1a). In injured F8-KO mice, the ΔFMT was significantly higher compared with WT (6 h *p* = 0.014; 12 h *p* = 0.024; 24 h *p* = 0.0001; 48 h *p* = 0.0005), and sham mice (12 h *p* = 0.029; 24 h *p* = 0.001) (Fig. 1b). No difference was found between WT and sham mice (data not shown). In addition, the fluorescent signals in the injured knees were significantly higher in F8-KO mice compared with WT (12 h *p* = 0.005; 24 h *p* = 0.015; 48 h *p* < 0.0001), and sham mice (24 h *p* = 0.026) (Fig. 1c), with no differences between WT and sham mice (data not shown). No differences in fluorescent signals of the contralateral knee were found (Fig. 1d). The optimal imaging time point was 24 h after induction: Here, the ΔFMT was significantly increased in F8-KO mice compared with WT (*p* = 0.0002) and sham (*p* = 0.007) mice (Fig. 1e).

**Fig. 2:**
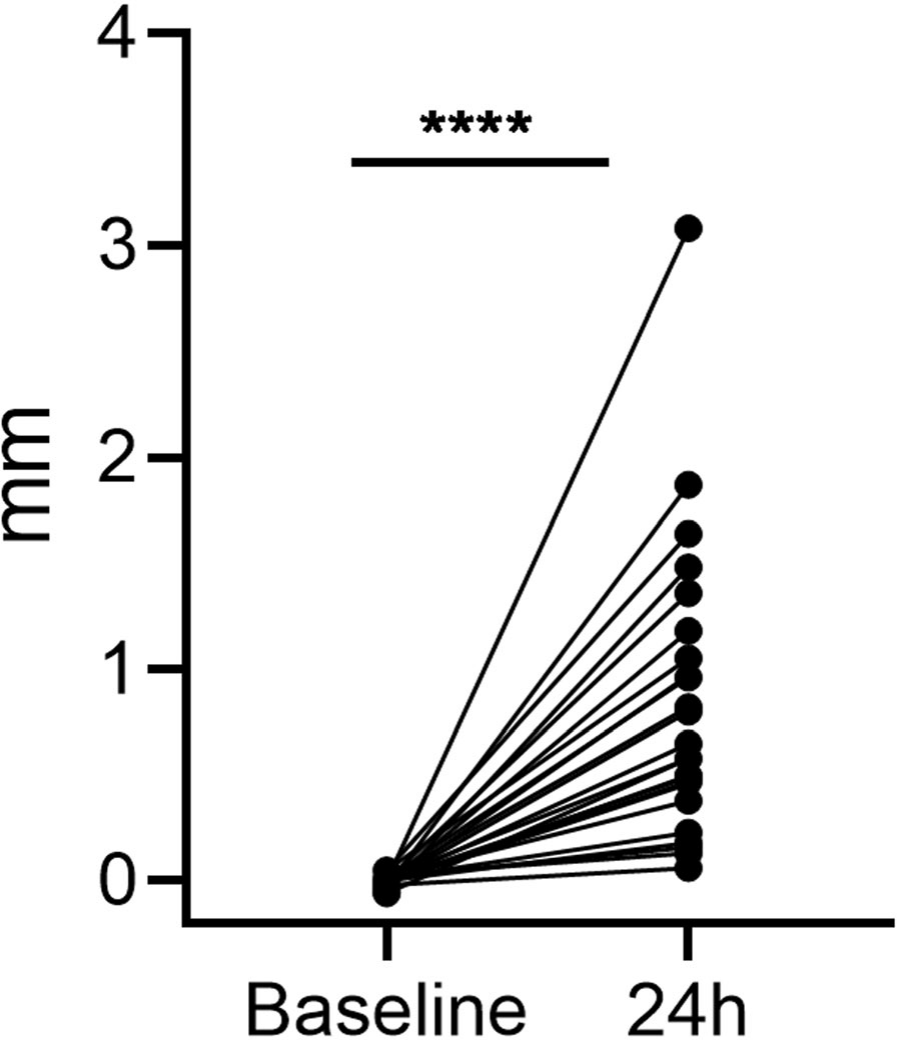
Joint diameter. In haemophilic mice, the difference in diameter of the induced versus the contralateral knee was significantly increased 24 h after induction. Statistical significance levels are marked: ****P < 0.0001

In the haemophilic mice, the induced joint bleed was also characterised by measuring the diameter of the joint prior to and 24 h after induction, demonstrating a significant increase in the diameter of the injured knee 24 h after induction (*p* < 0.0001) (Fig. 2).

### Induced joint bleed causes pathological bone remodelling and decreased bone volume fraction in F8-KO mice

**Fig. 3:**
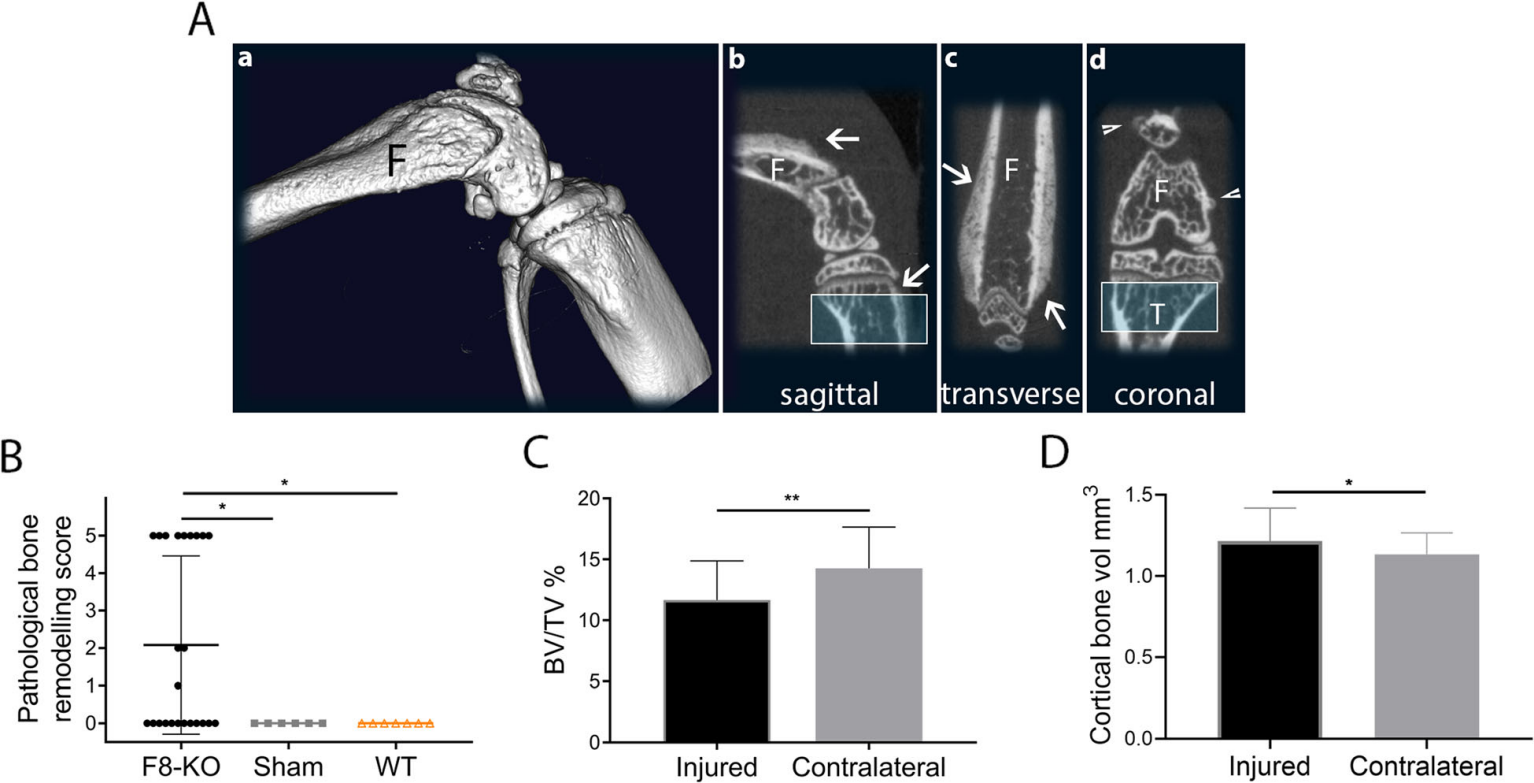
Haemophilic mice develop bone pathology 14 days after induced haemarthrosis. **a** Pathological bone remodelling in a F8-KO mouse. a 3D volume of an induced knee of a haemophilic mouse showing pathological bone remodelling. b, c, d: sagittal, transverse and coronal sections showing periosteal bone remodelling (arrows) and osteophytosis (arrowheads) on femur (F) and tibia (T). The blue box indicates the area used for bone volume fraction analysis on tibia. **b**: Half (12/24) of the F8-KO mice had a positive bone remodelling score, ranging from 1 to 5 and significantly higher than sham and WT mice. **c**: Bone volume fraction was significantly decreased in the proximal part of tibia of the injured knee compared to the contralateral. **d**: In addition, cortical bone volume was significantly increased in the injured knee compared to the contralateral. Statistical significance levels are marked: *P < 0.05, **P < 0.01. Error bars represent the standard deviation

Next, we examined the knee joints ex vivo with micro-CT 14 days post injury, a time point at which prominent bone changes are known to occur in this model [19, 28]. Representative images of a knee displaying extensive bone pathology is shown in Fig. 3A. Images were scored for presence of pathological bone remodelling on the cortical bone: Half (12/24) of the F8-KO mice developed pathological bone remodelling, with a score ranging from 1 to 5 (Fig. 3A, B). F8-KO mice had a significantly higher bone pathology score than WT (*p* = 0,031) and sham (p = 0,046) mice (Fig. 3B), in neither of which bone pathology was observed. In haemophilic mice, the proximal tibia of the injured knee had a significantly decreased bone volume fraction compared with the contralateral knee (*p* = 0.002, Fig. 3C), indicating loss of trabecular bone. In addition, the injured knee had an increased cortical bone volume (*p* = 0.03, Fig. 3D). In WT and sham mice, no differences between left and right knee were found (data not shown).

Further, the group of haemophilic mice that displayed pathological bone remodelling showed significant loss of trabecular bone compared with the group with no pathological bone remodelling (*p* < 0.0001, Supplemental Fig. 1).

### Bleeding characteristics on day 1 linked to subsequent development of bone pathology on day 14

**Fig. 4:**
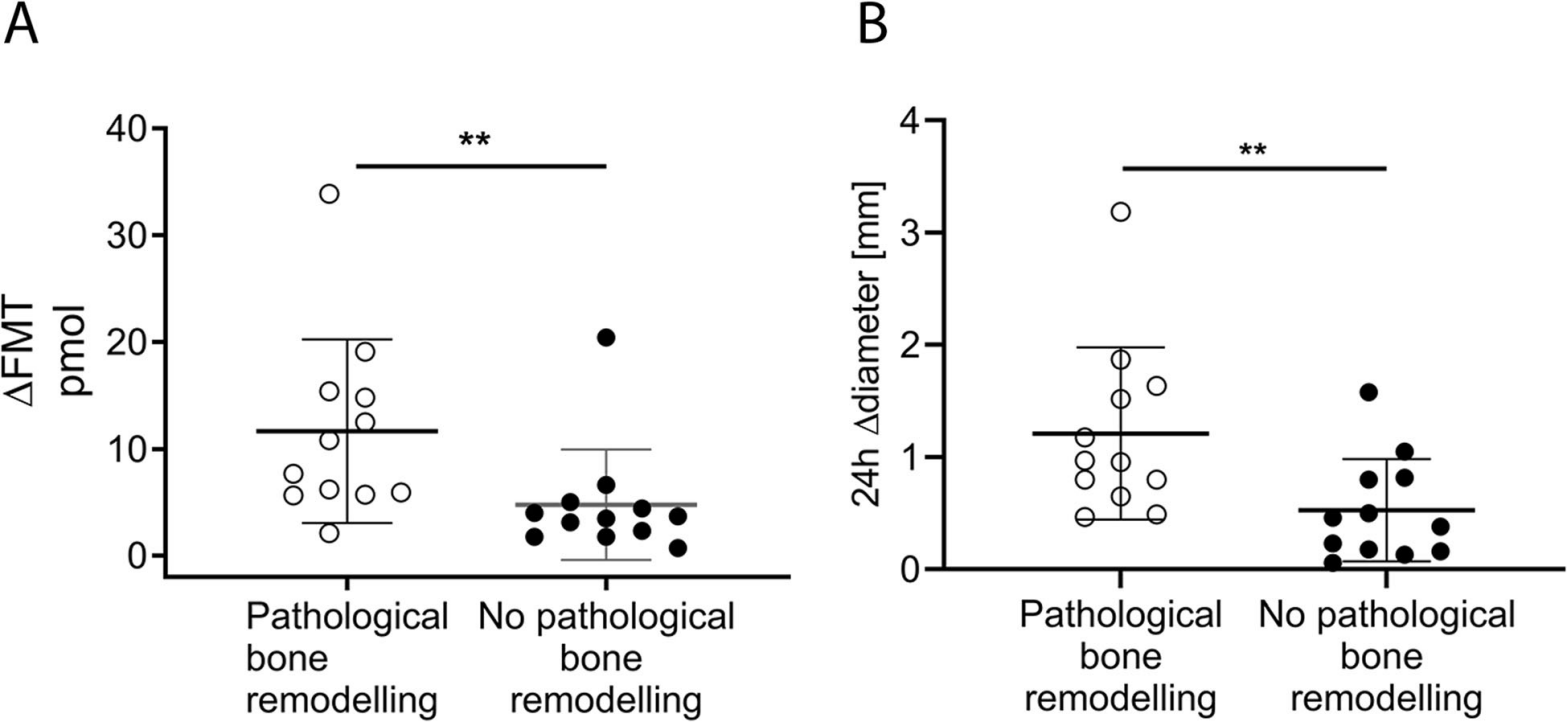
*In vivo *bleeding characteristics related to pathological bone remodelling: **a**: ΔFMT and joint diameter increase (**b**) 24 h after induced joint in F8-KO mice that developed pathological bone remodelling compared with those that did not (mean +/− standard deviation). Statistical significance levels are marked: **P < 0.01

**Fig. 5:**
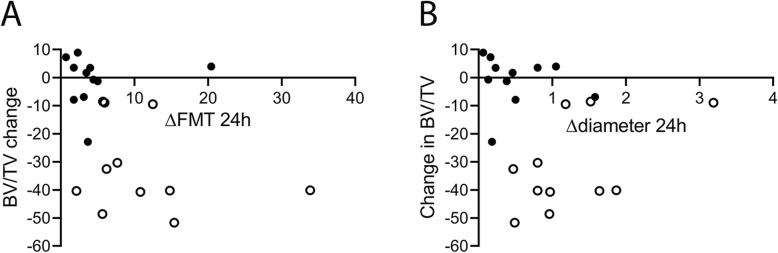
In vivo bleeding characteristics related to decrease in bone volume fraction: **a**: Correlation between the 24 h ΔFMT and the change in bone volume fraction (*p* = 0.053) (**b**): Correlation between the 24 h Δdiameter and the change in bone volume fraction (*p* = 0.34). White and black bullets represent mice with/without pathological bone remodelling, respectively

On day 14 post injury, 12 of 24 F8-KO mice had developed pathological bone remodelling. When dividing the F8-KO mice into two groups depending on whether bone pathology was present, the longitudinal difference in ΔFMT again peaked after 24 h. Here, F8-KO mice that developed pathological bone remodelling had a significantly higher ΔFMT (*p* = 0.004, Fig. 4a) and Δdiameter (*p* = 0.008, Fig. 4b) compared to F8-KO mice that did not develop pathological bone remodelling. In addition, we observed a borderline-significant correlation between the 24 h ΔFMT and the degree of trabecular bone loss (BV/TV) on Day 14 (*p* = 0.053, r^2^ = 0.17, Fig. 5a), whereas the Δdiameter did not correlate with the degree of trabecular bone loss (*p* = 0.35, r^2^ = 0.04, Fig. 5b).

### Effect of blood on the quantification of fluorescent intensities with FMT

**Fig. 6:**
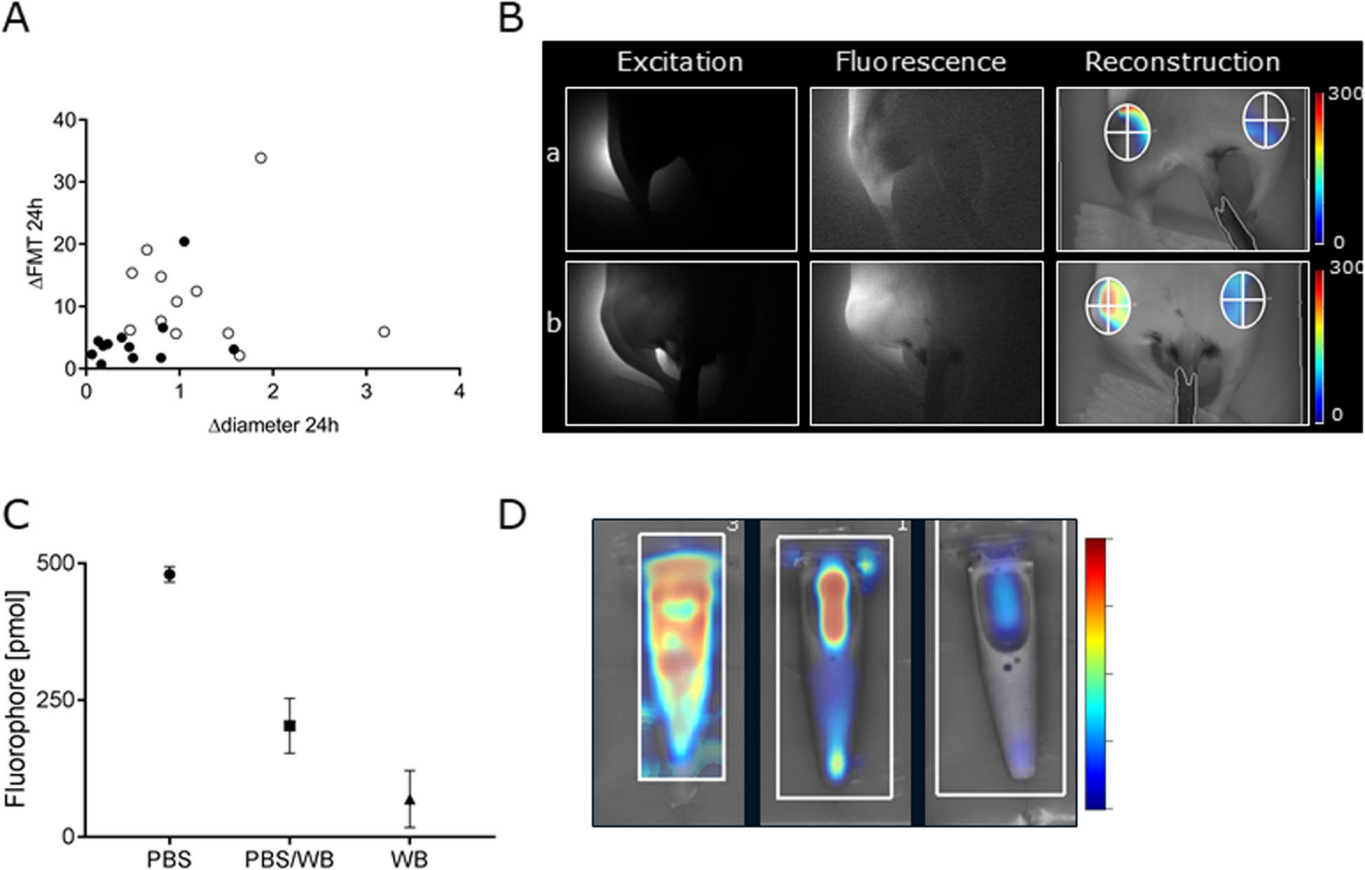
Attenuation of fluorescent signal by blood. **a**: Relative fluorescent signal in the injured knee, ΔFMT, depicted as function of joint diameter increase, Δdiameter, 24 h after induced joint bleed shows low ΔFMT at high Δdiameter. **b**: Excitation, fluorescence, and reconstructed image of a haemophilic mouse with a Δdiameter of 1.64 mm (a) and 0.49 mm (b) at 24 h. Colour bar shows intensity of signal. Note the attenuated signal in mouse a. **c**: Fluorescent signals measured with FMT after adding 500 pmol AngioSense750 to a 1.5 mL Eppendorf tube containing either 100% PBS; 50% v/v PBS and whole blood; or 100% whole blood (mean +/− standard deviation), as shown in (**d**)

To assess whether the fluorescent signal in the knees consistently reflected the amount of blood in the knee joint region, we correlated the ΔFMT with the Δdiameter (Fig. 6A) and found a non-significant correlation (*p* = 0.20, r^2^ = 0.07). This non-relation seemed to be driven by low ΔFMT values at high Δdiameters (Fig. 6A, B), becoming especially evident at Δdiameters above 1.2 mm (five mice). To address this phenomenon further, we tested how whole blood influenced the quantification of fluorescent signals with FMT by conducting an in vitro study. Fluorophore diluted in PBS yielded significantly higher fluorescent signal compared with dilution in blood (*p* = 0.003), whereas a trend was observed when compared with a 50% blood:PBS dilution (*p* = 0.23, Fig. 6C, D). Review of transillumination images revealed that increasing concentrations of blood in the sample were associated with increased photon attenuation, with almost complete extinction when fluorophore was spiked in whole blood only (Supplemental Fig. 2). However, if excluding the five F8-KO mice that had a Δdiameter above 1.2 mm at 24 h to account for this, it did not lead to any different conclusions with regards to the relations between 24 h ΔFMT and development of bone pathology (Figs. 3, 4); therefore, the animals were not excluded from the final analysis.

## Discussion

The present study is the first to apply optical in vivo imaging to characterise induced joint bleeds longitudinally in haemophilic mice. We demonstrate a link between the severity of the induced bleeds on the first day after induction, and the development of bone pathology 14 days later in individual mice.

In this study, we used FMT to characterise induced joint bleeds in vivo. We were able to distinguish the degree of bleeding observed in knee-injured haemophilic mice from that seen in WT and sham mice, based on the accumulation of fluorescent blood pool agent in the induced knee. However, we could not discriminate between the bleeding observed in knee-injured WT mice compared with sham mice. Previously, imaging of induced joint bleeds with contrast-enhanced micro-CT demonstrated that knee-injured WT mice develop a small bleeding (< 1 μl) upon knee injury, whereas no extravascular blood signal is seen in the knees of sham mice [19]. Thus, the sensitivity to detect small bleeds is lower with fluorescence imaging compared with micro-CT. This may partially be explained by the inferior resolution [29] that does not allow signal from intra-vascular blood (originating from large vessels in the knee joint region) to be distinguished from signal originating from extravascular blood in the knee joint region, thereby decreasing the signal to background ratio and making it very difficult to detect the small bleeds in WT mice.

Previously, near-infrared blood pool agents have been used successfully to characterise blood volume changes in relation to inflammation [30] but surprisingly, we observed that the fluorescent signals in the injured knee of haemophilic mice did not seem to reflect the amount of blood in the region in mice with a large increase in the diameter of the joint. Consequently, we compared the fluorescent signal in the knee with the corresponding change in diameter of that knee. Indeed, we found that large increases in knee diameter yielded a lower signal than observed in mice with a smaller increase in diameter. This could be related to the accumulation of haemoglobin, the main absorber of photons in the near-infrared range [31]. At near-infrared wavelengths, haemoglobin accounts for around half of the photon absorbance in a typical tissue containing 8% blood [32]. Consequently, the photon absorbance of haemoglobin is likely to play an even greater role in a knee joint region filled with blood, as seen in injured haemophilic mice. To explore this hypothesis, we conducted an in vitro study in which we saw increasing photon attenuation when the fluorescent probe was diluted in increasing proportions of whole blood. Similar findings are reported by Persigehl et al. [33]. For the current in vivo experiment, this implies that the fluorescent signals in the injured knees of haemophilic mice with large bleeds most likely have been underestimated. Also, this phenomenon could have led to an underestimation of the heterogeneity of the induced joint bleeds in haemophilic mice, as the impact of photon attenuation presumably would increase with increasing bleeding size. Possibly, this issue could be accounted for by using software optimised for quantifying fluorescent signals in optically-dark tissues [34]. This software was, however, not available for this study. However, as most of the induced bleed in haemophilic mice is resorbed within a week after induction (in house observations), imaging with FMT at later time points in this model will most likely not be influenced by signal attenuation by haemoglobin. In addition, imaging with activatable probes could provide information of molecular-expression patterns in the induced knee without the need for euthanising the animals [35]. Thus, FMT may be a powerful in vivo tool in future studies to unravel the mechanisms of blood-induced joint damage in haemophilia and at the same time reduce the number of animals needed.

Interestingly, both the relative fluorescence as well as the change in diameter of the injured knee on Day 1 were linked to the presence of cortical pathological bone remodelling 14 days later. The mechanism behind cortical pathological bone remodelling in haemophilic arthropathy is currently unknown, but it is possible that large bleeds in haemophilia A mice lead to a delayed but high and sustained level of thrombin in the joint, as seen in haemophilia B mice [36]. As osteoblasts are activated by thrombin [37], this could explain why cortical bone remodelling was associated with larger bleeds in this study, but would need to be investigated further. Intriguingly, the development of pathological bone remodelling in F8-KO mice was highly dichotomous; in general, either no pathological bone remodelling was seen, or it was present on all bone surfaces. Previously, it has been shown that the pathological bone remodelling score gradually increases during the first 7 days after induced haemarthrosis [38], suggesting that mediators disrupting physiological bone turnover will eventually affect the entire joint.

In addition, we also found a nearly-significant correlation between the severity of the induced bleed on Day 1 – as described by the relative fluorescent signal - and the degree of trabecular bone loss in the proximal tibia on Day 14, which has not been shown previously to our knowledge. Osteopenia is a well-described comorbidity in haemophilia that is related to the severity of arthropathy in the patient, although many factors such as HIV- and hepatitis infection as well as physical activity level may also be part of the cause [39, 40]. In addition, there is growing evidence that inflammation, mediated by tumour necrosis factor alpha (TNF-α), plays a central role in blood-induced bone loss [41, 42]. In patients with haemarthrosis there is a correlation between the haemoglobin concentration in the joint and the level of TNF-α expression in the joint [41], which could explain the relation between the severity of bleeding and the loss of trabecular bone observed in this study.

## Conclusion

In conclusion, we demonstrate for the first time that induced joint bleeds in haemophilic mice can be described longitudinally in vivo with FMT, although signal attenuation by haemoglobin should be accounted for. Further, we show that the severity of joint bleeding just 1 day after induction is a strong determinant for subsequent development of bone pathology in individual mice. These findings support further investigation into early clinically-relevant biomarkers for subsequent joint damage.

## Supplementary information

**Additional file 1: Figure S1.** Change in bone volume fraction in haemophilic mice with/without pathological cortical bone remodelling. Haemophilic mice displaying pathological bone remodelling had a significantly decreased bone volume fraction in the tibia of the injured knee. Statistical significance levels are marked: ****P < 0.0001.

**Additional file 2: Figure S2.** Transillumination images of Eppendorf tubes with/without blood. Excitation- (left column) and emission (right column) transillumination FMT images of Eppendorf tubes containing 500 pmol AngioSense added to either 1.5 mL PBS (top row), 0.75 mL whole blood and 0.75 mL PBS (middle row), or 1.5 mL whole blood. Increased photon attenuation is seen with increased proportions of whole blood in the Eppendorf tube.

**Additional file 3: Table S1.** Imaging block contents

## Data Availability

The datasets supporting the conclusions of this article are available in the Figshare repository at: 10.6084/m9.figshare.9124823.v2.

## Abbreviations

BV/TV: Bone volumetric density
CT: Computed tomography
FMT: Fluorescence molecular tomography
FVIII: Factor VIII
KO: Knock out
PBS: Phosphate buffered saline
VOI: Volume of interest
WB: Whole blood
WT: Wild-type
