# Supplementary figures and images for: In vivo fluorescence molecular tomography of induced haemarthrosis in haemophilic mice: link between bleeding characteristics and development of bone pathology
Source: BMC Musculoskelet Disord. 2020 Apr 14;21:241. doi: 10.1186/s12891-020-03267-5 (PMC7158129; doi:10.1186/s12891-020-03267-5)

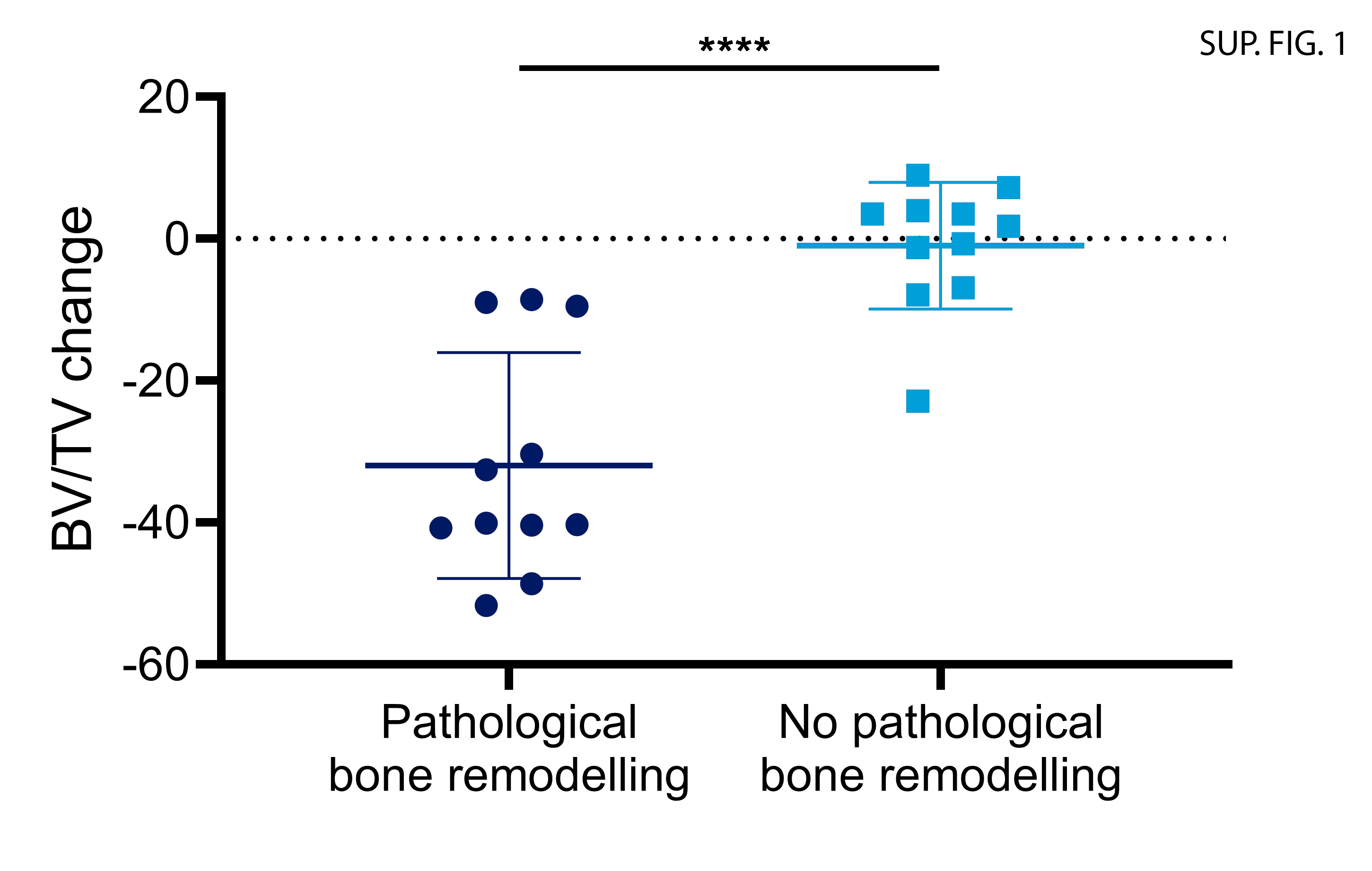

Supplement: Supplementary file 1 — Additional file 1: Figure S1. Change in bone volume fraction in haemophilic mice with/without pathological cortical bone remodelling. Haemophilic mice displaying pathological bone remodelling had a significantly decreased bone volume fraction in the tibia of the injured knee. Statistical significance levels are marked: ****P < 0.0001. [file 12891_2020_3267_MOESM1_ESM.tif]

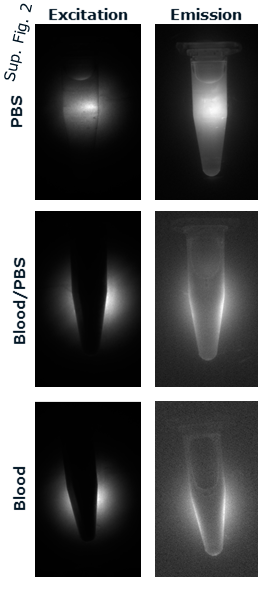

Supplement: Supplementary file 2 — Additional file 2: Figure S2. Transillumination images of Eppendorf tubes with/without blood. Excitation- (left column) and emission (right column) transillumination FMT images of Eppendorf tubes containing 500 pmol AngioSense added to either 1.5 mL PBS (top row), 0.75 mL whole blood and 0.75 mL PBS (middle row), or 1.5 mL whole blood. Increased photon attenuation is seen with increased proportions of whole blood in the Eppendorf tube. [file 12891_2020_3267_MOESM2_ESM.tif]
