# Supplementary material for: In vivo fluorescence molecular tomography of induced haemarthrosis in haemophilic mice: link between bleeding characteristics and development of bone pathology
Source: BMC Musculoskelet Disord. 2020 Apr 14;21:241. doi: 10.1186/s12891-020-03267-5 (PMC7158129; doi:10.1186/s12891-020-03267-5)
Supplement: Supplementary file 3 — Additional file 3: Table S1. Imaging block contents [file 12891_2020_3267_MOESM3_ESM.docx]

Imaging block contents

| Description | Vendor/Cat# | Amount per batch |
| --- | --- | --- |
| Titanium (IV) oxide | Aldrich/ 232 033 | 250mg |
| 1% Black india ink | Higgins/ 44201 | 0.32ml |
| Ethanol | N/A | 1.82ml |
| TAP Clear-Lite Casting Resin | TAP/Clear-lite casting resin | 180g |
| MEKP liquid catalyst | TAP/MEKP liquid catalyst | 2.5ml |
